# Supplementary material for: Vitamin D Modified DSS-Induced Colitis in Mice via STING Signaling Pathway
Source: Biology (Basel). 2025 Jun 18;14(6):715. doi: 10.3390/biology14060715 (PMC12190092; doi:10.3390/biology14060715)
Supplement: Supplementary file 1 [file biology-14-00715-s001.zip › Supplementary Materials S1: 16s-report of gut microbiota/Groups/taxanomyBar/taxon_hist/without_unknown/top10/Family.groups.distribution.pdf]

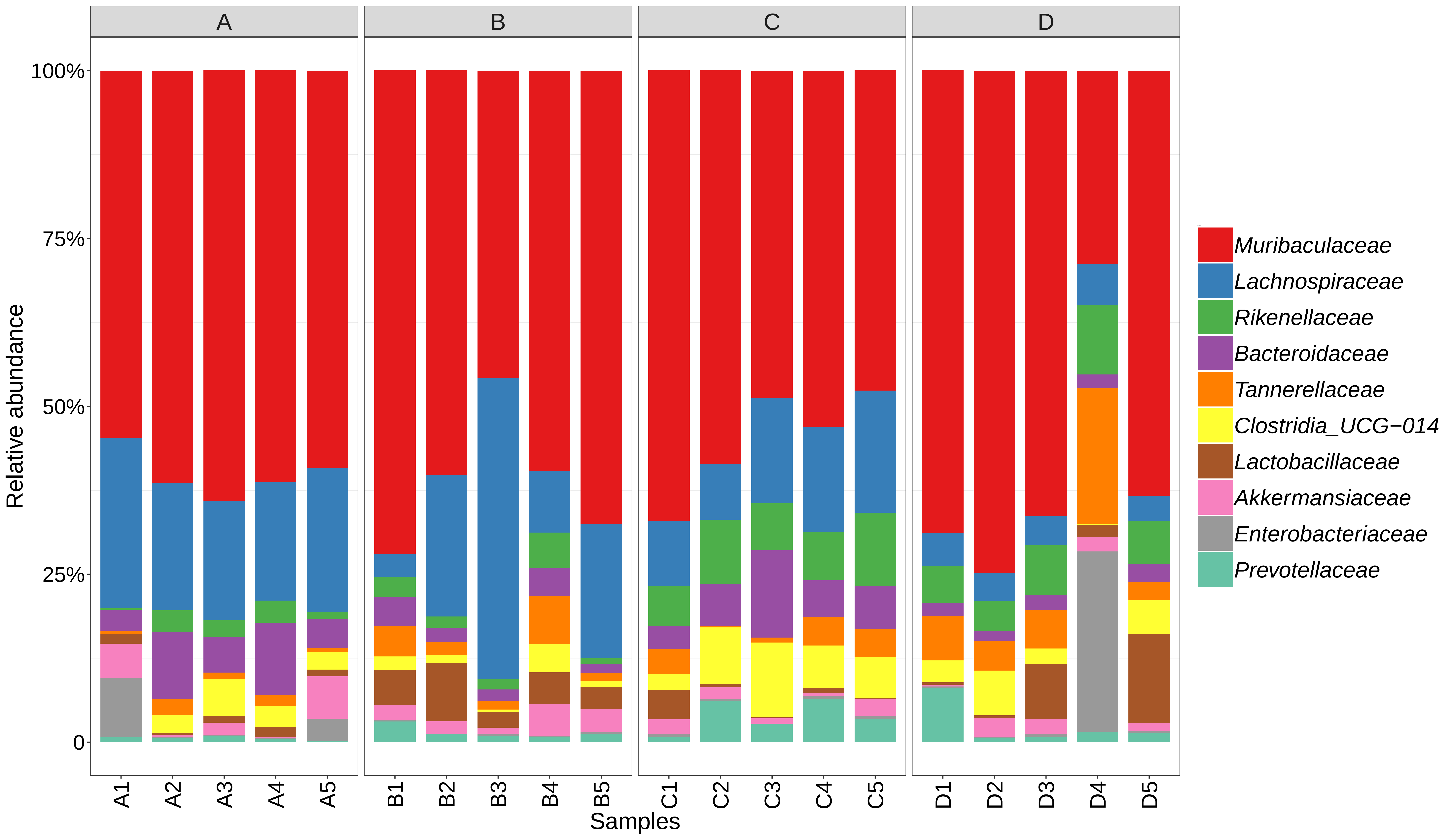
A
B
C
D
100%
75%
50%
25%
0
Relative abundance
A1
A2
A3
A4
A5
B1
B2
B3
B4
B5
C1
C2
C3
C4
C5
D1
D2
D3
D4
D5
Samples
Muribaculaceae
Lachnospiraceae
Rikenellaceae
Bacteroidaceae
Tannerellaceae
Clostridia_UCG−014
Lactobacillaceae
Akkermansiaceae
Enterobacteriaceae
Prevotellaceae
